# Carotid atherosclerosis and right ventricular diastolic dysfunction in a sample of hypertensive Nigerian patients

**DOI:** 10.3325/cmj.2013.54.555

**Published:** 2013-12

**Authors:** Adeseye A. Akintunde, Philip B. Adebayo, Ademola A. Aremu, Oladimeji G. Opadijo

**Affiliations:** 1 Department of Medicine, LAUTECH Teaching Hospital, Ogbomoso, Nigeria; 3 Department of Radiology, LAUTECH Teaching Hospital, Ogbomoso, Nigeria; 2 Goshen Heart Clinic, Osogbo, Nigeria

## Abstract

**Aim:**

To determine the association of carotid atherosclerosis and right ventricular diastolic dysfunction (DD) among treated hypertensive Nigerian patients.

**Methods:**

This was a single center cross-sectional study performed at the Cardiology Clinic of LAUTECH Teaching Hospital, Ogbomoso, Nigeria between January and December 2012. The study included 122 hypertensive Nigerians (mean age, 57.3 ± 14.7 years, 36.9% women). Patients’ clinical, demographic, and echocardiographic parameters were obtained. Diastolic dysfunction was assessed with the trans-tricuspid Doppler flow.

**Results:**

Patients with DD were significantly older than those with normal diastolic function. Mean and maximum carotid intima media thickness measurements were significantly higher among patients with right ventricular DD than in those with normal diastolic function. Mean systolic blood pressure (148.3 ± 31.9 vs 128.0 ± 2.8 mm Hg, *P* = 0.049) and interventricular septal thickness in diastole (12.8 ± 2.3 vs 11.6 ± 2.8mm, *P* = 0.048) were significantly higher and tricuspid annular pulmonary systolic excursion (33.6 ± 4.9 vs 23.0 ± 4.2 mm, *P* = 0.035) was significantly lower in patients with right ventricular DD than in those with normal diastolic function. Carotid intima media thickness measurements were correlated with early trans-tricuspid Doppler flow and early transtricuspid diastolic flow/late right atrial transtricupsid diastolic flow ratio.

**Conclusion:**

Right ventricular DD in hypertensive patients was significantly correlated with increased carotid atherosclerosis. Carotid intima media thickness measurements may therefore be a surrogate marker for DD in hypertensive subjects.

Systemic hypertension has been shown to be associated with right ventricular abnormalities in both morphology and function. Right ventricular diastolic dysfunction (DD) may be an early indicator of hypertensive heart disease accompanying left ventricular DD (1-3). It can be diagnosed by various means including the ratio of early and late trans-tricuspid Doppler inflow velocities and tissue Doppler velocities.

Atherosclerosis is the underlying factor for many cases of cardiovascular morbidity and mortality (4). A useful surrogate marker for carotid atherosclerosis is carotid intima media thickness (5). This parameter reflects the severity of thickening/atherosclerosis of the major arteries and is directly linked with the severity of hypertension and target organ damage associated with hypertension (2,4). DD is a typical part of the cardiovascular risk spectrum with increased risk for accelerating progressive cardiac dysfunction (6). However, it is still not known whether carotid intima media thickness, which has been shown to correlate with many conventional cardiovascular risk factors, is associated with right ventricular DD, especially among Africans (7). This study aims to determine the association between right ventricular DD and carotid intima media thickness in a sample of Nigerian hypertensive patients.

## Patients and methods

This was a single center cross-sectional study performed at the Cardiology Clinic of LAUTECH Teaching Hospital, Ogbomoso, Nigeria. One hundred and twenty two hypertensive patients were consecutively recruited for this study. The study was conducted between January and December 2012. We obtained clinical and demographic parameters, including age, sex, weight, height, and body mass index.

The study included hypertensive participants who were on treatment and had been diagnosed according to the standardized criteria (8). Hypertension was defined as persistent elevated sitting blood pressure >140/90 mm Hg (the patients had to be sitting for at least five minutes). At the time of the study some participants had normal blood pressure due to the applied therapy. All participants underwent echocardiography according to the recommendation of the American Society of Echocardiography (9). We excluded those who had been diagnosed with diabetes mellitus, chronic kidney disease, liver disease, were pregnant, were on statin treatment or refused to give their consent to participate in the study.

Echocardiography and B Mode Carotid Doppler were performed using General Electric Ultrasound Machine (LOGIQPro 6) with 3.5MHz and 9 MHz probes, respectively. The parameters obtained in the echocardiography included posterior wall thickness in diastole (PWTd), interventricular septal thickness in diastole (IVSd), left ventricular end diastolic dimension (LVDD), left ventricular end systolic dimension (LVSD), and right ventricular dimension (RVD). Left ventricular mass and left ventricular mass index were calculated using the Devereux modified ASE cube formula (6). Right ventricular diastolic function was assessed using the trans-tricuspid Doppler flow velocity. The early (E wave) and late (A wave) trans-tricuspid wave velocity were determined by putting the Doppler sampling volume at the ventricular end of the tricuspid valve. Right ventricular diastolic dysfunction was defined as Tricuspid E/A ratio <1. The right ventricular dimension was taken in the left parasternal long axis in the mid-ventricular section.

The carotid intima media thickness was measured in common carotid artery, with the participant lying down, neck extended and head slightly turned in the direction opposite to the carotid artery being examined. A 10-mm longitudinal section located at a distance of 1-2 cm from the bifurcation was studied and measurements were performed in the distal walls along an axis perpendicular to the artery, to establish two lines: the intima-media interface and the media-adventitia interface. Six measurements were obtained in each carotid artery, and the average mean and maximum values were recorded (5,7).

Statistical analysis was done using the SPSS 18.0 (SPSS Inc., Chicago, IL, USA). Quantitative data are presented as means ± standard deviation and qualitative data as frequency and percentages. Group comparison was done using *t* test and χ^2^ test. Pearson correlation statistics was done to show the association between indices of carotid atherosclerosis and that of right ventricular diastolic function. *P* < 0.05 was set as the significance level. The study conforms to the Protocol of Helsinki and ethical approval was obtained from the institutional ethics research committee. Written and informed consent was obtained from all participants.

## Results

The mean age of the study participants was 57.3 ± 14.7 years (Table 1). Of 122 patients, 45 were women (36.9%). About half of the study participants had their blood pressure controlled, ie had blood pressure ≤140/90 mm Hg. Fifty-eight patients (47.5%) had evidence of right ventricular DD. The mean systolic blood pressure of patients with right ventricular DD was significantly higher than of patients with normal right ventricular diastolic function (148.3 ± 31.9 vs 128.0 ± 2.8 mm Hg, *P* = 0.049).

The mean and maximum carotid intima media thickness measurements were significantly higher in participants with right ventricular DD than in those with normal diastolic function (Table 2). Participants with right ventricular diastolic dysfunction were significantly older than those with normal right ventricular diastolic function (62.8 ± 13.8 vs 53.2 ± 15.1 years, *P* = 0.013). Patients with right ventricular DD had significantly higher mean systolic blood pressure, interventricular septal thickness in the diastole (12.8 ± 2.3 vs 11.6 ± 2.8mm, *P* = 0.048), and posterior wall thickness in the diastole (11.8 ± 2.4 vs 11.3 ± 5.2mm, *P* = 0.781) than patients with normal diastolic function. Patients with normal diastolic function had significantly higher tricuspid annular pulmonary systolic excursion (TAPSE), an indicator of right ventricular systolic function (33.6 ± 4.9 vs 23.0 ± 4.2 mm, *P* = 0.035) (Table 2).

Carotid intima media thickness was correlated with the early trans-tricuspid diastolic Doppler flow and the ratio of early trans-tricuspid diastolic flow and late right atrial systole (TE/TA) (Table 3). Other right ventricular parameters such as TA, pulmonary systolic velocities, pressure gradient, right ventricular internal diastolic dimension, and TAPSE were not significantly correlated with the carotid intima media thickness measurements. The exception was that the peak pulmonary arterial systolic velocities correlated significantly with mean carotid intima media thickness in the left carotid artery and the pulmonary pressure gradient significantly correlated with the mean carotid intima media thickness in the right carotid artery (Table 3).

## Discussion

Our study found that right ventricular DD in hypertensive patients was significantly correlated with increased carotid atherosclerosis. Systemic hypertension has been associated with many long term complications including systolic and diastolic heart failure, stroke, and myocardial infarction (10-12). Diastolic dysfunction associated with hypertension, especially of the left ventricle, has been linked with increasing mortality and morbidity (13,14). Diastolic heart failure (or heart failure with preserved ejection fraction) has been reported to have a higher risk and worse prognosis than heart failure with reduced ejection fraction (13,15). There is evidence that systemic hypertension is often followed by similar affectation of the right ventricle as well as the left ventricle in function and in morphology (1).

Carotid atherosclerosis, reflected by the carotid intima media thickness is additional evidence of increasing predisposition to complications due to stroke and myocardial infarction. This study provides an additional set of information linking carotid atherosclerosis with right ventricular DD among treated hypertensive Nigerians. We noted that all carotid intima media thickness measurements were correlated with the early trans-tricuspid right ventricular filling pressures and also with the ratio of the early and late right ventricular diastolic inflow velocity. This study also revealed that carotid intima media thickness measurements were significantly higher in patients with right ventricular DD than in those with normal right ventricular diastolic function. In addition, mean interventricular septal thickness which is the shared part with the left ventricle was significantly greater than the posterior wall in hypertensive patients with right ventricular DD than in those with normal diastolic function. We postulate that the impact of an additional insult from the right ventricular DD on the interventricular septum might be responsible for this. These two groups were similar in body mass index distribution and the impact of obesity is therefore almost excluded. Hypertensive patients in this study with right ventricular DD had significantly higher mean carotid intima media thickness measurements than hypertensive patients with normal right ventricular diastolic function as evaluated by the trans-tricuspid E/A ratio. Hypertensive patients with right ventricular diastolic dysfunction were significantly older than hypertensive subjects with normal right ventricular diastolic function.

Plausible explanations for the apparent involvement of the right ventricle in systemic hypertension are suggested. However, many of them are not well elucidated. The mechanisms of ventricular interaction are unknown but may relate to restriction of ventricular filling by the pericardium (16), although most studies have assessed only the effect of right ventricular volume expansion on left ventricular function (17) rather than vice versa. Other explanations include enhanced sympathetic tone, increased delivery of blood borne vasoconstrictor substances, or abnormal local release of vasoactive factors acting on both the greater and the lesser circulation (18).

Tricuspid annular pulmonary systolic excursion is a relative good estimate of right ventricular systolic function (19). Although both were within the normal limits, we noted that the right ventricular systolic function of patients with DD was significantly worse than of patients with normal diastolic function. This may be a consequence of associated progressive decline in right ventricular systolic function with increasing right ventricular DD in association with carotid atherosclerosis, which this study was not able to detect. This may be a new finding; we believe that tissue Doppler and right ventricular fractional area change may be a better instrument to delineate right ventricular function.

This study therefore confirms the results of other studies that have shown that right ventricular diastolic dysfunction is a marker of adverse effects as it parallel blood pressure driven left ventricular diastolic remodeling (20). The relationship between left ventricular DD and carotid stiffness or atherosclerosis has been better described among Caucasians (21-23). However, because of the systemic nature of most of the pathogenetic linkages associated with left ventricular DD, there is ample evidence that right ventricle is equally affected in hypertension. Hypertensive patients with DD had a higher risk of carotid atherosclerosis and consequently increased cardiovascular risk as revealed by a significantly higher mean carotid intima media thickness, which is a surrogate marker of cardiovascular burden. Apart from this, left ventricular mass and wall chamber dimensions were significantly higher among those with increased atherosclerosis, which further points to a seemingly higher cardiovascular burden.

This study has some limitations. It is a hospital based study and the result cannot be directly extrapolated to the general population. The trans-tricuspid Doppler flow, which was used as a measure of DD can be affected by preload and its impact could not be assessed. Also, this was a cross-sectional study and therefore it cannot investigate causal relations between carotid atherosclerosis and right ventricular DD. A well designed prospective study will be necessary in that regard. Furthermore, our Center does not have tissue Doppler, which could have possibly revealed more patients with right ventricular diastolic dysfunction.

In conclusion, this study revealed that right ventricular DD in hypertensive patients was significantly correlated with carotid atherosclerosis. Further studies are necessary to reveal the timing and other determinants of biventricular diastolic involvement in hypertensive patients.

## 

**Table 1:** Clinical and echocardiographic parameters of study participants (n = 122)*

Variable	Values, mean ± standard deviation or No (%)
Age, years	57.3 ± 14.7
Women	45 (36.9)
SBP (mmHg)	137.3 ± 29.1
DBP (mmHg)	78.0 ± 13.8
BP<140/90 mm Hg	58 (47.5)
BMI (kg/m^2^)	26.6 ± 6.3
LVDD (mm)	48.1 ± 8.7
EF (%)	60.8 ± 15.4
IVSd (mm)	12.1 ± 2.7
PWTd (mm)	11.5 ± 3.8
AOD (mm)	31.5 ± 4.4
LAD (mm)	39.7 ± 7.7
RT CIMT 1 (mm)	1.11 ± 0.51
RT CIMT 2 (mm)	0.90 ± 0.38
LT CIMT 1 (mm)	1.14 ± 0.47
LT CIMT 2 (mm)	0.95 ± 0.33
Subjects with RVDD	58(47.5)

*Abbreviations: SBP – systolic blood pressure; DBP – diastolic blood pressure; BMI – body mass index; LVDD – left ventricular end diastolic dimension; EF – ejection fraction; IVSd – interventricular septal thickness in diastole; PWTd – posterior wall thickness in diastole; AOD – aortic root dimension; LAD – left atrial dimension; RT CIMT 1 – maximum right carotid intima media thickness; RT CIMT 2 – mean right carotid intima media thickness; LT CIMT 1 – maximum left carotid intima media thickness; LT CIMT 2 – mean left carotid intima media thickness.

**Table 2:** Clinical, echocardiographic, and carotid artery intima media thickness parameters of hypertensive participants with right ventricular (RV) diastolic dysfunction and those with normal diastolic function (mean ± standard deviation)*

Variable	Patients with RV diastolic dysfunction (n = 58)	Patients with normal RV diastolic function (n = 64)	*P* (*t* test)
Age (years)	62.8 ± 13.8	53.2 ± 15.1	0.013^†^
SBP (mmHg)	148.3 ± 31.9	128.0 ± 2.8	0.049^†^
DBP (mmHg)	80.3 ± 17.9	74.0 ± 8.5	0.852
LVDD (mm)	44.0 ± 5.5	46.3 ± 7.6	0.219
LVSD (mm)	29.5 ± 6.9	31.7 ± 10.3	0.273
IVSd (mm)	12.8 ± 2.3	11.6 ± 2.8	0.048^†^
PWTd (mm)	11.8 ± 2.4	11.3 ± 5.2	0.781
LAD (mm)	39.2 ± 6.3	39.6 ± 6.2	0.829
AOD (mm)	32.2 ± 4.5	31.0 ± 3.6	0.489
TAPSE (mm)	23.0 ± 4.2	33.6 ± 4.9	0.035^†^
LVMI (g/m^2^)	57.1 ± 40.2	55.5 ± 18.2	0.036^†^
RT CIMT 1 (mm)	1.29 ± 0.51	1.09 ± 0.57	0.016^†^
RT CIMT 2 (mm)	0.98 ± 0.44	0.76 ± 0.30	0.017^†^
LT CIMT 1 (mm)	1.33 ± 0.46	1.10 ± 0.49	0.009^†^
LT CIMT 2 (mm)	0.97 ± 0.22	0.84 ± 0.37	0.023^†^

*Abbreviations: SBP – systolic blood pressure; DBP – diastolic blood pressure; IVSd – interventricular septal thickness in diastole; PWTd – posterior wall thickness in diastole; AOD – aortic root dimension; LAD – left atrial dimension; RT CIMT 1 – maximum right carotid intima media thickness; RT CIMT 2 – mean right carotid intima media thickness; LT CIMT 1 – maximum left carotid intima media thickness; LT CIMT 2 – mean left carotid intima media thickness; LVSD – left ventricular end systolic dimension; TAPSE – tricuspid annular pulmonary systolic excursion; LVMI – left ventricular mass index.

†Significant.

**Table 3:** Correlation statistics of right ventricular diastolic indices to carotid intimal media thickness measurements*

Variable	RT CIMT 1	RT CIMT 2	LT CIMT 1	LT CIMT2
TE	-0.191^†^	-0.199^†^	-0.228^†^	-0.219^†^
	0.045	0.041	0.016	0.026
TA	-0.009	-0.053	0.051	0.081
	0.940	0.649	0.655	0.489
TE/TA	-0.238^†^	-0.318^†^	-0.277^†^	-0.294^†^
	0.035	0.005	0.013	0.011
PPSV	-0.058	-0.177	-0.131	-0.198^†^
	0.549	0.069	0.171	0.044
PPPGR	-0.008	-0.192^†^	0.022	-0.139
	0.930	0.048	0.817	0.158
TAPSE	-0.082	-0.210	-0.108	-0.224
	0.551	0.138	0.433	0.122
RVD	0.12	0.084	-0.003	-0.047
	0.896	0.386	0.977	0.635

*Abbreviations: TE – early trans-tricuspid diastolic flow; TA – late right atrial systole; TE/TA – ratio of TE/TA. PPSV – peak pulmonary systolic velocity; PPPGR – peak pulmonary pressure gradient; TAPSE – tricuspid annular pulmonary systolic excursion; RVD – right ventricular dimension; RT CIMT 1 – maximum right carotid intima media thickness; RT CIMT 2 – mean right carotid intima media thickness; LT CIMT 1 – maximum left carotid intima media thickness; LT CIMT 2 – mean left carotid intima media thickness.

^†^Significant correlation statistics. N.B the correlation coefficients are on the top segment of each cell in the table, while the *P* value is at the bottom of the cell.
